# Preoperative Magnetic Resonance Imaging Results Are Concordant with Pathology Staging in Rectal Cancer

**DOI:** 10.3390/medicina62020328

**Published:** 2026-02-06

**Authors:** Dursun Burak Ozdemir, Serdar Senol, Mirsad Yalcinkaya

**Affiliations:** 1 Department of General Surgery, Samsun City Hospital, 55090 Samsun, Turkey; 2 Department of General Surgery, Samsun University, Samsun City Hospital, 55090 Samsun, Turkey; serdarardaduru@gmail.com; 3 Department of Radiology, Samsun University, Samsun City Hospital, 55090 Samsun, Turkey; mirsadyalcinkaya@hotmail.com

**Keywords:** rectal neoplasms, magnetic resonance imaging, neoadjuvant therapy, pathology, tumor staging

## Abstract

*Background and Objectives:* Magnetic resonance imaging (MRI) is the gold standard for rectal cancer staging; however, its reliability after neoadjuvant therapy (NAT) remains controversial due to treatment-induced tissue changes. This study aimed to compare preoperative MRI findings with postoperative pathologic results in rectal cancer patients following NAT and to assess MRI reliability across clinical subgroups. *Materials and Methods:* This single-center retrospective study included 47 adult patients with locally advanced rectal adenocarcinoma who received NAT followed by elective rectal resection, with preoperative pelvic MRI and postoperative pathology results. Clinical features, MRI (T stage, N stage, and circumferential resection margin [CRM]), and pathologic staging were recorded. The endpoints were defined as concordance (via kappa coefficients) and predictive performance (via ROC analysis). *Results:* Among 47 patients (mean age 63.5 ± 9.4 years; 80.9% male), MRI demonstrated slight concordance with pathology for the T stage (kappa = 0.178, *p* = 0.028) and moderate concordance with the N stage (kappa = 0.489, *p* < 0.001), but not for CRM (*p* = 0.154). Subgroup analyses revealed significant concordance for N stage across most subgroups, with lower rectal tumors showing significant agreement for all three parameters. ROC analysis demonstrated significant predictive value for the N stage (AUC = 0.776, *p* = 0.002) with 88.6% specificity, while the T stage and CRM showed non-significant discriminatory performance. *Conclusions:* Post-NAT MRI demonstrates moderate reliability relative to pathology, particularly for N staging, and may have even better utility in specific subgroups stratified for sex, NAT type, and tumor site.

## 1. Introduction

Colorectal cancer is a common type of cancer, particularly among males [1,2], and a leading cause of cancer-related mortality, with rectal adenocarcinoma comprising about 30–35% of colorectal malignancies [1,3,4]. Management includes conservative and surgical interventions ranging from less invasive approaches to aggressive resection, and treatment decisions are challenging due to proximity to critical structures like the anal sphincter and pelvic nerves, and higher risk of local recurrence compared to colon cancers [5,6]. For locally advanced disease (cT3-4 or N+), standard treatment involves neoadjuvant chemoradiotherapy followed by total mesorectal excision (TME), which is reliably effective [7,8]. However, neoadjuvant therapy (NAT) may cause treatment-related fibrosis, mucinous changes, and downstaging, which complicate restaging and decision-making [9,10].

Magnetic resonance imaging (MRI) is the gold standard for primary staging and restaging after neoadjuvant treatments, aiding in tumor localization, T and N staging, circumferential resection margin (CRM) assessment, and extramural vascular invasion (EMVI) detection [7,11]. Studies have also demonstrated utility in identifying metastatic lymph nodes [12,13]. However, the reliability of MRI after NAT remains debated, with available concordance rates varying widely. For instance, some studies show low agreement for T staging (47–62%) and N staging (64–77%) due to challenges in distinguishing fibrosis from residual tumor [14,15]. Overstaging of superficial tumors (T0, T1 and T2) and underestimation of nodal disease are also common, running the risk for overtreatment or missed opportunities for organ preservation [16,17]. Diagnostic accuracy studies report conflicting results, and there has been limited focus on CRM and EMVI assessment. The variability in MRI protocols is another critical factor that might prevent generalization of relationships from center to center [18,19]. The limited data on stratified analyses may be another concern, as tumor location, NAT type, or patient-related factors might influence outcomes. A recent meta-analysis by Zager et al. [20], including 23 studies, confirmed limited concordance between post-NAT MRI and pathology staging with substantial heterogeneity across centers, underscoring the need for continued investigation of factors influencing MRI reliability.

This study aims to compare preoperative MRI results with postoperative pathologic findings in rectal cancer patients after neoadjuvant chemoradiotherapy and assess whether preoperative MRI is a reliable modality for staging.

## 2. Materials and Methods

### 2.1. Study Design and Setting

This research was designed as a single-center, retrospective study and conducted at the Department of General Surgery of Samsun Training and Research Hospital, Samsun, Turkiye.

### 2.2. Study Population and Eligibility Criteria

The study population consisted of adult patients who had a histopathological diagnosis of rectal adenocarcinoma, received neoadjuvant therapy, underwent elective rectal resection, and had both preoperative pelvic MRI staging and postoperative pathological evaluation available. Patients younger than 18 years, those with recurrent or metastatic disease at presentation, individuals with incomplete radiological or pathological data, patients in whom MRI was performed before neoadjuvant therapy, those undergoing emergency surgery, and patients with histological subtypes other than adenocarcinoma were excluded.

**Figure 1:**
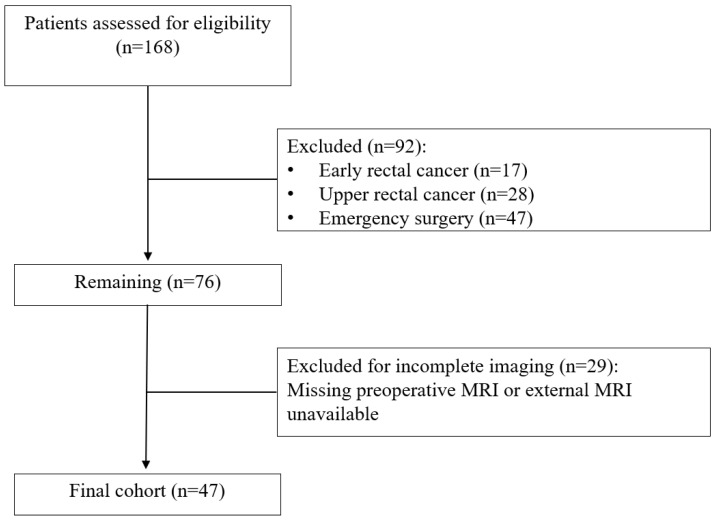
Flowchart of the study.

Patient flow through the study is presented in Figure 1. Of 168 patients who underwent rectal resection for rectal cancer during the study period, 92 were excluded: 17 had early-stage rectal cancer, 28 had upper rectal tumors, and 47 underwent emergency surgery. Among the remaining 76 patients, 29 were excluded due to missing preoperative MRI or external imaging that could not be retrieved, yielding a final study cohort of 47 patients.

This study was conducted in accordance with the ethical standards of the Declaration of Helsinki and its amendments. Ethical approval was obtained from the Non-Interventional Clinical Research Ethics Committee of Samsun University (Approval date: 12 November 2025, decision no: 2025/22/23).

### 2.3. Data Collection

Data were retrospectively collected from the hospital’s electronic medical records system, radiology archives, and pathology reports. The variables included demographic information such as age and sex; clinical characteristics including the ASA score, which was recorded according to the American Society of Anesthesiologists classification [21]; and NAT details including NAT type (long-course or short-course; long-course defined as 45–50.4 Gy radiotherapy combined with chemotherapy, and short-course defined as 25 Gy delivered in five fractions) [22] and NAT regimen (standard versus total NAT, with total NAT defined as induction or consolidation chemotherapy combined with chemoradiotherapy).

Time-related parameters included the interval between the completion of NAT and MRI, the interval between NAT completion and surgery, and the interval between MRI and surgery. Tumor characteristics included tumor location categorized as upper, middle, or lower rectum based on the distance from the anal verge (upper: 10–15 cm, middle: 5–10 cm, lower: 0–5 cm) [23]. Surgical details were extracted from operative records and included the type of procedure performed, documented as low anterior resection (LAR), abdominoperineal resection (APR), or their laparoscopic equivalents.

### 2.4. MRI Acquisition and Interpretation

Preoperative MRI findings included the T stage according to the AJCC 8th edition TNM system [24], the N stage, and circumferential resection margin (CRM) status. CRM was recorded as positive if the tumor or any associated tumor component was located ≤1 mm from the mesorectal fascia [25]. Radiological CRM assessment was performed by measuring the shortest distance from the tumor, suspicious lymph nodes, tumor deposits, or extramural vascular invasion to the mesorectal fascia, which appears as a thin hypointense line on T2-weighted images, with measurements taken perpendicular to the tumor axis in axial or oblique-axial planes. Pelvic magnetic resonance imaging was performed using two 1.5-Tesla MRI systems available at our institution (Siemens and GE Healthcare). An identical standardized rectal cancer MRI protocol was applied to both scanners. The imaging protocol included axial non-fat-suppressed T1-weighted sequences and non-fat-suppressed T2-weighted sequences in axial, coronal, and sagittal planes. All sequences were acquired with a slice thickness of 3 mm. Diffusion-weighted imaging (DWI) was performed in the axial plane using b-values of 0, 50, and 800 s/mm^2^, and apparent diffusion coefficient (ADC) maps were automatically generated. Following the administration of intravenous contrast, fat-suppressed T1-weighted dynamic contrast-enhanced sequences were obtained in the axial plane. The dynamic study included pre-contrast, arterial, venous, and delayed phases, and subtraction images were generated. Lymph node assessment on MRI was based on morphological criteria, including size (short-axis diameter ≥ 5 mm), irregular borders, heterogeneous signal intensity, and restricted diffusion on DWI, consistent with established guidelines for rectal cancer nodal staging.

All MRI evaluations were performed by a single radiologist with 11 years of experience in gastrointestinal and pelvic MRI. The radiologist was blinded to postoperative pathology results during image assessment, with access only to anonymized imaging data. As a single reader performed all interpretations, inter-reader reliability assessment was not applicable

### 2.5. Pathological Assessment

Postoperative pathology reports provided data on pathological T stage, N stage, and CRM status. CRM positivity was defined as tumor extension of ≤1 mm on pathological assessment. Pathological analyses were performed by expert gastrointestinal pathologists on total mesorectal excision specimens according to the AJCC 8th edition criteria. All patient data were anonymized and stored in a secure password-protected database. Pathological staging was performed using the ypTNM system according to AJCC 8th edition criteria. All TME specimens were examined using a standardized reporting protocol that included macroscopic tumor dimensions, histological subtype, tumor grade, tumor regression score, pathological T and N staging, tumor budding, and lymphovascular invasion status. Pathologists were not blinded to preoperative MRI findings, as is standard clinical practice. Lymph node retrieval followed institutional standards.

### 2.6. Statistical Analysis

Sample size justification was assessed post hoc using the kappa coefficients reported by Akgül et al. (effect size = 0.367). With 95% confidence level (α = 0.05) and 90% power, the minimum required sample size was 41 patients, which was exceeded by our cohort of 47 patients. Sample size calculation was performed using PASS 11 software (NCSS, LLC, Kaysville, UT, USA) with the ‘Kappa Test for Agreement Between Two Raters’ module.

Statistical analyses were conducted using IBM SPSS version 27.0 (IBM Corp., Armonk, NY, USA) and MedCalc Statistical Software version 23.1.7 (MedCalc Software Ltd., Ostend, Belgium). Two-tailed *p*-values of less than 0.05 were considered statistically significant. For the normality check, the Shapiro–Wilk test was used. Descriptive statistics were presented using mean ± standard deviation for normally distributed continuous variables, median (25th percentile–75th percentile) for non-normally distributed continuous variables, and frequency (percentage) for categorical variables. Concordance between MRI and pathology findings was evaluated using Cohen’s weighted kappa coefficients with linear weights. Kappa coefficients were interpreted as “poor” for negative values, “slight” for 0.000–0.200, “fair” for 0.201–0.400, “moderate” for 0.401–0.600, “substantial” for 0.601–0.800, and “almost perfect” for 0.801–1.000 [26]. In instances where small sample sizes resulted in asymptotic confidence intervals exceeding theoretical bounds (−1 to 1), intervals were truncated to the valid range for interpretability while retaining the underlying asymptotic estimation method. The prediction performance of the MRI was assessed using the receiver operating characteristic (ROC) curve analysis. For ROC curve analysis, staging variables were dichotomized based on clinically meaningful thresholds: the T stage was categorized as T3–T4 versus T0–T2 (distinguishing locally advanced from early-stage tumors relevant for neoadjuvant therapy decisions), the N stage as N1–N2 versus N0 (distinguishing nodal positivity relevant for prognosis and treatment planning), and CRM as positive (≤1 mm) versus negative (>1 mm) (distinguishing high-risk margins associated with local recurrence). Confidence intervals for diagnostic performance metrics (sensitivity, specificity, likelihood ratios, predictive values) were calculated using standard methods (Clopper-Pearson for proportions, log method for likelihood ratios, logit method for predictive values) as implemented in MedCalc Statistical Software. Given the extremely low event rate for positive CRM (*n* = 2, 4.3%), AUC estimates for CRM should be interpreted with caution and are reported for descriptive purposes only. All analyses were performed on a complete-case dataset with no missing values for the primary endpoints (T stage, N stage, and CRM). No imputation methods were applied.

## 3. Results

A total of 47 patients were included in the study, with a mean age of 63.53 ± 9.35 years. The majority were male (38 patients, 80.9%). Detailed demographic, clinical, and imaging characteristics, including ASA scores, time intervals between NAT, MRI, and surgery, NAT type/regimen, tumor location, surgical procedures, MRI T and N stages, MRI CRM status, pathology T and N stages, and pathology CRM status, are summarized in Table 1.

The concordance between MRI and pathology findings for the T stage, N stage, and CRM is presented in Table 2. Overall examination of the patients revealed slight concordance for T stage (kappa = 0.178, 95% CI 0.000–0.357, *p* = 0.028) and moderate concordance for the N stage (kappa = 0.489, 95% CI 0.229–0.749, *p* < 0.001), but not for CRM (*p* = 0.154). When stratified by sex, fair concordance was observed in males for T stage (kappa = 0.216, 95% CI 0.028–0.405, *p* = 0.016) and moderate concordance for the N stage (kappa = 0.470, 95% CI 0.188–0.751, *p* = 0.001). In females, concordance was significant (substantial) only for the N stage (kappa = 0.609, 95% CI −0.057–1.274, *p* = 0.047). In ASA III patients, substantial concordance was found for the N stage (kappa = 0.625, 95% CI 0.322–0.929, *p* < 0.001) and moderate concordance was found for CRM (kappa = 0.465, 95% CI −0.133–1.064, *p* = 0.008).

By type of NAT, concordance in long course NAT (the great majority of patients) was significant for the N stage (kappa = 0.483, 95% CI 0.220–0.746, *p* < 0.001). Among recipients of standard NAT regimen, concordance was significant for the T stage (kappa = 0.170, 95% CI −0.093–0.433, *p* = 0.035) and CRM (kappa = 1.000, 95% CI 1.000–1.000, *p* = 0.005). In the total NAT group, concordance was significant for the T stage (kappa = 0.201, 95% CI −0.007–0.409, *p* = 0.037) and N stage (kappa = 0.458, 95% CI 0.185–0.731, *p* < 0.001). Stratified by tumor location, in middle rectum tumors, concordance was significant for the N stage (kappa = 0.519, 95% CI 0.228–0.811, *p* < 0.001). In lower rectum tumors, concordance was significant for the T stage (kappa = 0.308, 95% CI −0.063–0.678, *p* = 0.045), N stage (kappa = 0.353, 95% CI −0.074–0.780, *p* = 0.026), and CRM (kappa = 0.621, 95% CI −0.035–1.277, *p* = 0.026).

**Figure 2:**
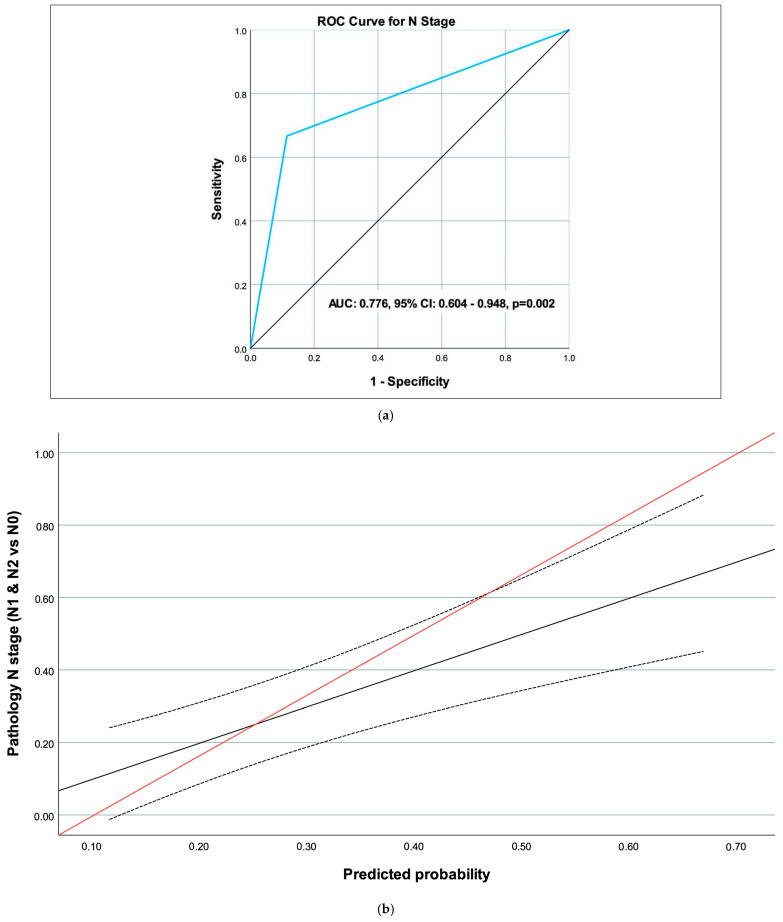
(**a**) ROC curve of magnetic resonance imaging (MRI) to predict the N stage. (**b**) Calibration curve of magnetic resonance imaging (MRI) to predict the N stage (black line: calibration curve, dotted lines: confidence interval for calibration curve, red line: reference line).

**Figure 3:**
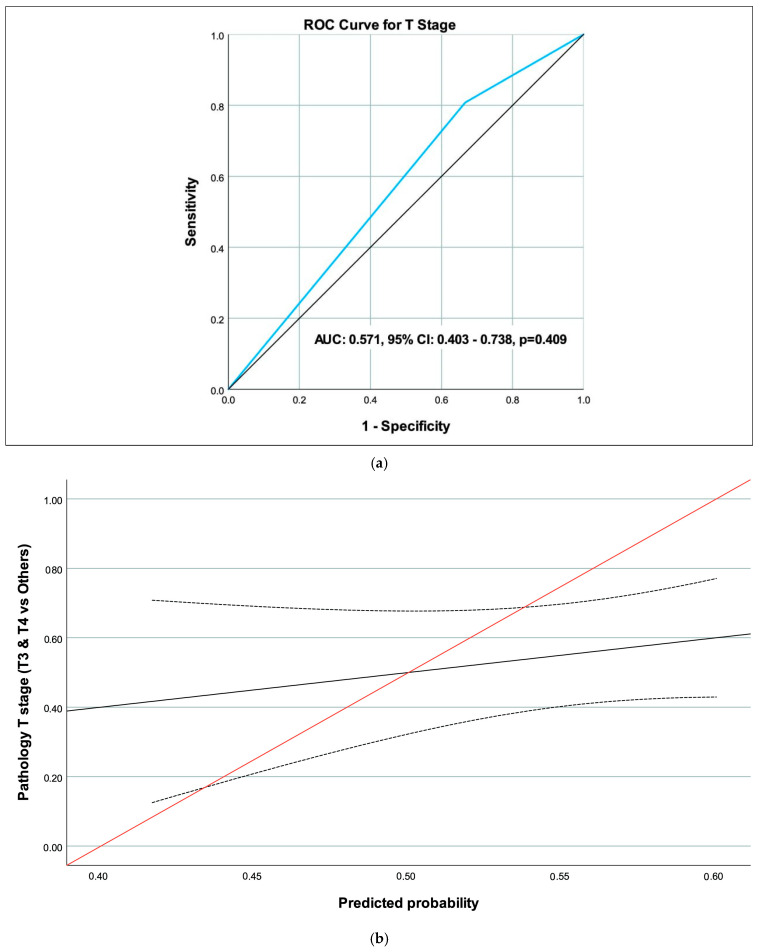
(**a**) ROC curve of magnetic resonance imaging (MRI) to predict the T stage. (**b**) Calibration curve of magnetic resonance imaging (MRI) to predict the N stage (black line: calibration curve, dotted lines: confidence interval for calibration curve, red line: reference line).

**Figure 4:**
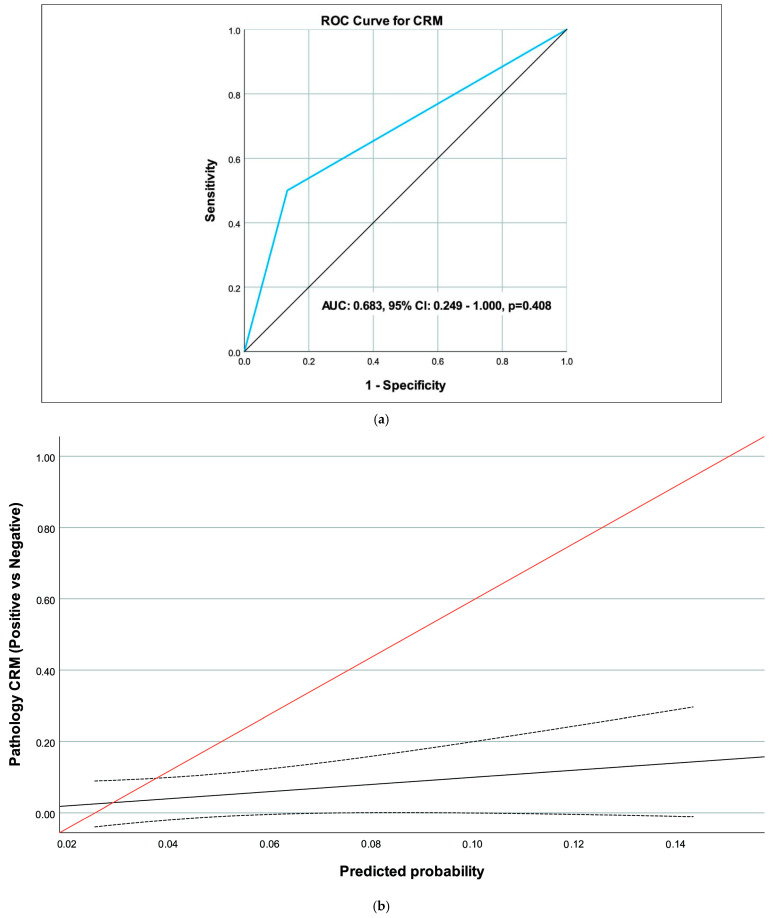
(**a**) ROC curve of magnetic resonance imaging (MRI) to predict circumferential resection margin (CRM). (**b**) Calibration curve of magnetic resonance imaging (MRI) to predict circumferential resection margin (CRM) (black line: calibration curve, dotted lines: confidence interval for calibration curve, red line: reference line).

The performance of MRI in predicting pathology findings, based on ROC curve analysis for the T stage (T3 & T4), N stage (N1 & N2), and CRM (Positive), is summarized in Table 3. For the N stage, the area under the curve (AUC) was 0.776 (95% CI 0.604–0.948, *p* = 0.002), indicating significant predictive value, with a sensitivity of 66.7% (95% CI 34.9–90.1) and a specificity of 88.6% (95% CI 73.3–96.8) (Table 4) (Figure 2a,b). For the T stage (Figure 3a,b) and CRM (Figure 4a,b), the AUC values were 0.571 (95% CI 0.403–0.738, *p* = 0.409) and 0.683 (95% CI 0.249–1.000, *p* = 0.408), respectively, showing no significant predictive performance (Table 3). The CRM analysis was limited by sparse positive events (*n* = 2), and these AUC estimates should be interpreted with caution.

## 4. Discussion

We reveal that preoperative MRI demonstrated moderate reliability in correlating with postoperative pathologic findings among rectal cancer patients who had received NAT, with better performance for nodal (N) staging. Significant concordance was observed overall for the T stage and N stage, though not for CRM status. Subgroup analyses demonstrated important variations that may be crucial in the clinical setting. Data for females showed significance only for the N stage, long-course NAT correlated significantly with the N stage, and lower rectal tumors seemed to show the best concordance values, significant for T, N, and CRM stages. ROC analyses were promising for N staging, particularly for specificity, but sensitivity was moderate. These findings suggest that MRI has considerable utility in nodal assessment but limitations in T staging and CRM prediction after NAT. Our findings align with a 2024 meta-analysis by Zager et al. [20], which demonstrated similar limited concordance for T-staging and moderate reliability for N-staging across 23 pooled studies. While our results do not exceed the existing evidence base, they contribute single-center data with detailed subgroup analyses that may inform future investigations into patient and tumor factors influencing MRI accuracy.

A key focus of this investigation was the concordance between post-NAT MRI and pathologic T staging, which is crucial for guiding surgical decisions and assessing tumor downstaging. Rectal cancer management relies heavily on accurate T staging to differentiate superficial from advanced tumors, influencing choices between sphincter-preserving surgery and more radical approaches [1]. Prior studies have noted challenges in post-treatment T staging due to therapy-induced changes, such as fibrosis mimicking residual tumor [3]. In our cohort, overall concordance for the T stage was slight, with significant agreement in males, the standard NAT group, the total NAT group, and lower rectal tumors. However, ROC performance was non-significant, despite a sensitivity of 80.8% due to low specificity (33.3%) for T3/T4, indicating a tendency toward overstaging. This aligns with prior data showing that post-chemoradiotherapy MRI showed 52% accuracy for the T stage, primarily due to overstaging of T0, T1 and T2 tumors due to inflammatory changes [5]. Similarly, Johnston et al. reported 82% histopathological agreement for the T stage on post-treatment MRI, but noted downstaging in only 41% of cases (possibly in relation to fibrotic changes); however, the sample size was small [4]. Our findings show higher sensitivity but lower specificity, possibly reflecting the overrepresentation of middle rectal tumors (76.6%) in our data. Neoadjuvant chemoradiotherapy induces apoptosis, necrosis, and fibrotic replacement in tumor tissue, altering signal intensity on T2-weighted images and leading to overestimation of invasion depth [7]. Thus, fibrotic changes could explain overstaging as well as inflammatory cell infiltration [8]. We demonstrate that lower tumors can be more reliably assessed with pre-intervention MRI, which is a novel aspect despite the limited sample size. From a clinical perspective, T-stage overstaging may lead to unnecessarily aggressive surgical approaches or exclusion of patients from organ-preservation strategies. Clinicians should therefore interpret post-NAT MRI T-staging cautiously, recognizing that apparent tumor extension may represent treatment-related changes rather than viable tumors.

Nodal staging emerged as a strength of MRI based on both overall and sub-group results for concordance and ROC analyses –high specificity (88.6%) and NPV (88.6%). Accurate nodal evaluation is vital, as persistent nodal disease is associated with poorer prognosis and may necessitate extended lymphadenectomy [9,10]. The predictive metrics determined in our study are relatively higher compared to most studies, which might be due to various reasons, including patient characteristics as well as the MRI methodology/evaluation. Yuval et al. described 71.8% nodal accuracy on restaging MRI, with variability due to reader experience and signal heterogeneity [7]. Capirci et al. utilized a PET/MRI fusion analysis in 81 patients and found 79% specificity for complete response but only 45% sensitivity for nodal status, attributing low sensitivity to post-radiation inflammation masking micrometastases [8]. In a retrospective cohort of 270 patients, nodal restaging accuracy was 66.1%, lower than our 74.5% N0 concordance [12]. Fibrotic changes and hyalinization may also lead to signal changes that obscure occult metastases [14]. Our high specificity suggests MRI excels in ruling out nodal involvement.

CRM assessment post-NAT can be critical for predicting local recurrence [16]. Our study found non-significant overall concordance and discriminatory performance (86.7% specificity, but low sensitivity, 50.0%) for positive CRM. Significant agreements were present in subgroups, such as in the standard NAT regimen and lower rectal tumors. Margins can be blurred by desmoplastic reactions, which impact CRM results [17]. Popita et al. reported 81.3% nodal accuracy, with CRM concordance at 78.2%, similar to our overall data [16]. A meta-analysis of six studies (916 patients) showed mrTRG1 specificity of 93.5% but again noted CRM limitations due to edema [11]. In lower tumors, our stronger concordance may stem from better visualization of sphincter involvement [27]. NAT can induce vascular permeability changes and mucin production, causing signals that mimic tumor extension to the mesorectal fascia [28]. Inflammatory mediators like cytokines might exacerbate this impact, leading to false positives [29].

Despite these insights, our study has notable limitations that warrant description. The retrospective design introduces selection bias, and generalizability is limited due to both a small sample size and the single-center design. The sample size of 47 patients represents a significant limitation that restricts the precision of our estimates and the generalizability of our findings to broader populations. This is particularly relevant for subgroup analyses, where cell sizes were frequently below 15 patients, yielding unstable concordance estimates with wide confidence intervals. Subgroup analyses were underpowered, particularly for categories with fewer than 15 patients, increasing the risk of both false-positive and false-negative findings. The number of lymph nodes examined per specimen was not systematically recorded, which limits our ability to fully characterize the reference standard for nodal staging assessment. The extremely low prevalence of pathologically positive CRM (4.3%, *n* = 2) severely limited the precision and generalizability of CRM-related analyses, including ROC curve performance metrics. Also, we only included patients undergoing elective resection after NAT, potentially excluding patients with better outcomes. The dominance of middle rectal tumors (76.6%) also skews overall data to the normative results of this group. Absence of upper rectal tumors further restricts applicability to proximal lesions. Time intervals were rather large, with a median of 77 days from NAT to surgery and 17 days from MRI to surgery. This might limit the comparability of MRI and pathology results. Additionally, this study did not incorporate FDG-PET/CT or PET/MRI, which are increasingly utilized for response assessment and nodal evaluation after NAT in rectal cancer and may provide complementary information to conventional MRI. Furthermore, the limited sample size increases the risk of model overfitting in our ROC analyses, potentially yielding overly optimistic AUC estimates, particularly for N-stage prediction. The absence of external validation or bootstrapping methods means these performance metrics should be interpreted as exploratory and may not generalize to independent cohorts.

## 5. Conclusions

In conclusion, this retrospective cohort study demonstrated that post-NAT MRI has slight concordance with pathology for T-staging (kappa = 0.178) and moderate concordance for N-staging (kappa = 0.489), consistent with the existing literature. MRI demonstrated high specificity (88.6%) for ruling out nodal disease, which may support clinical decision-making regarding organ preservation strategies. However, the limited reliability of T-stage and CRM assessment, combined with a tendency toward overstaging, necessitates cautious interpretation in clinical practice. Subgroup variations observed in this study are exploratory and require validation in larger cohorts. Future prospective multicenter studies with larger sample sizes are essential to identify patient and tumor characteristics that may predict MRI accuracy and to develop more reliable restaging strategies.

## Figures and Tables

**Table 1:** Summary of variables.

Age	63.53 ± 9.35
Sex	
Male	38 (80.9%)
Female	9 (19.1%)
ASA score	
ASA II	24 (51.1%)
ASA III	23 (48.9%)
Time between NAT and MRI, days	54.02 ± 21.92
Time between NAT and operation, days	77 (64–91)
Time between MRI and operation, days	17 (9–47)
Type of NAT	
Long course	45 (95.7%)
Short course	2 (4.3%)
Neoadjuvant therapy	
Standard	8 (17.0%)
Total	39 (83.0%)
Tumor location	
Upper	0 (0.0%)
Middle	36 (76.6%)
Lower	11 (23.4%)
Operation	
LAR	23 (48.9%)
APR	2 (4.3%)
Laparoscopic LAR	20 (42.6%)
Laparoscopic APR	2 (4.3%)
MRI T stage	
T0	1 (2.1%)
T1	4 (8.5%)
T2	7 (14.9%)
T3	30 (63.8%)
T4	5 (10.6%)
MRI N stage	
N0	35 (74.5%)
N1	9 (19.1%)
N2	3 (6.4%)
MRI CRM	
Positive	7 (14.9%)
Negative	40 (85.1%)
Pathology T stage	
T0	9 (19.1%)
T1	3 (6.4%)
T2	9 (19.1%)
T3	17 (36.2%)
T4	9 (19.1%)
Pathology N stage	
N0	35 (74.5%)
N1	9 (19.1%)
N2	3 (6.4%)
Pathology CRM	
Positive	2 (4.3%)
Negative	45 (95.7%)

Descriptive statistics presented using mean ± standard deviation for normally distributed continuous variables, median (25th–75th percentile) for non-normally distributed ones, and frequency (percentage) for categorical variables. Abbreviations: APR: Abdominoperineal Resection, ASA: American Society of Anesthesiologists, CRM: Circumferential Resection Margin, LAR: Low Anterior Resection, MRI: Magnetic Resonance Imaging, NAT: Neoadjuvant Therapy, ROC: Receiver Operating Characteristic, TME: Total Mesorectal Excision.

**Table 2:** Concordance between MRI and pathology findings.

		T Stage			N Stage			CRM	
	Agreement	Kappa Coefficient (95% CI)	*p*	Agreement	Kappa Coefficient (95% CI)	*p*	Agreement	Kappa Coefficient (95% CI)	*p*
All patients ^a^	34.0%	0.178 (0.000–0.357)	**0.028**	78.7%	0.489 (0.229–0.749)	**<0.001**	85.1%	0.167 (−0.188–0.522)	0.154
Sex									
Male	36.8%	0.216 (0.028–0.405)	**0.016**	76.3%	0.470 (0.188–0.751)	**0.001**	86.8%	0.228 (−0.216–0.672)	0.113
Female	22.2%	−0.161 (−0.585–0.263)	0.511	88.9%	0.609 (-0.057–1.274) ^b^	**0.047**	77.8%	0.000 (0.000–0.000)	1.000
ASA score									
ASA II	25.0%	0.155 (−0.073–0.384)	0.163	75.0%	0.300 (−0.106–0.706)	0.102	79.2%	−0.071 (−0.188–0.046)	0.648
ASA III	43.5%	0.180 (−0.078–0.438)	0.112	82.6%	0.625 (0.322–0.929)	**<0.001**	91.3%	0.465 (−0.133–1.064) ^b^	**0.008**
Type of NAT									
Long course	31.1%	0.139 (−0.038–0.315)	0.095	77.8%	0.483 (0.220–0.746)	**<0.001**	84.4%	−0.040 (−0.108–0.029)	0.692
Short course	100.0%	1.000 (1.000–1.000)	0.157	100.0%	N/A		100.0%	1.000 (1.000–1.000)	0.157
Neoadjuvant therapy									
Standard	37.5%	0.170 (-0.093–0.433)	**0.035**	100.0%	N/A		100.0%	1.000 (1.000–1.000)	**0.005**
Total	33.3%	0.201 (−0.007–0.409)	**0.037**	74.4%	0.458 (0.185–0.731)	**<0.001**	82.1%	−0.046 (−0.126–0.034)	0.666
Tumor location									
Middle	33.3%	0.140 (−0.060–0.340)	0.143	77.8%	0.519 (0.228–0.811)	**<0.001**	83.3%	−0.049 (−0.130–0.033)	0.684
Lower	36.4%	0.308 (−0.063–0.678)	**0.045**	90.9%	0.353 (−0.074–0.780)	**0.026**	90.9%	0.621 (−0.035–1.277) ^b^	**0.026**

N/A: Non-applicable because each variable in each pair has less than two valid categories (all patients were “N0”). ^a^ Statistical findings of all patients are our primary endpoint, and subgroup analysis results are exploratory. ^b^ In small samples, asymptotic kappa confidence intervals may exceed theoretical bounds. For interpretability, confidence intervals were truncated to the valid range [−1, 1] and the underlying asymptotic method was retained. Abbreviations: ASA: American Society of Anesthesiologists, CI: Confidence Interval, CRM: Circumferential Resection Margin, MRI: Magnetic Resonance Imaging, N/A: Not Applicable, NAT: Neoadjuvant Therapy.

**Table 3:** Performance of MRI to predict pathology findings, ROC curve analysis.

	T Stage	N Stage	CRM
State category	T3 & T4	N1 & N2	Positive
Sensitivity (95% CI)	80.8 (60.6–93.4)	66.7 (34.9–90.1)	50.0 (1.3–98.7)
Specificity (95% CI)	33.3 (14.6–57.0)	88.6 (73.3–96.8)	86.7 (73.2–94.9)
PLR (95% CI)	1.21 (0.85–1.73)	5.83 (2.13–15.94)	3.75 (0.78–18.09)
NLR (95% CI)	0.58 (0.21–1.56)	0.38 (0.17–0.85)	0.58 (0.14–2.32)
PPV (95% CI)	60.0 (51.2–68.2)	66.7 (42.3–84.5)	14.3 (3.3–44.6)
NPV (95% CI)	58.3 (34.1–79.1)	88.6 (77.5–94.6)	97.5 (90.7–99.4)
AUC (95% CI)	0.571 (0.403–0.738)	0.776 (0.604–0.948)	0.683 (0.249–1.000)
*p*	0.409	**0.002**	0.408

Abbreviations; AUC: Area Under ROC Curve, CI: Confidence Interval, NLR: Negative Likelihood Ratio, NPV: Negative Predictive Value, PLR: Positive Likelihood Ratio, PPV: Positive Predictive Value, ROC: Receiver Operating Characteristic.

**Table 4:** Contingency tables for dichotomized data.

		**Pathology T stage**	
		**T3 & T4**	**T0 & T1 & T2**
MRI T stage	T3 & T4	21 (TP = 80.8%)	14 (FP = 66.7%)
	T0 & T1 & T2	5 (FN = 19.2%)	7 (TN = 33.3%)
		**Pathology N stage**	
		**N1 & N2**	**N0**
MRI N stage	N2 & N1	8 (TP = 66.7%)	4 (FP = 11.4%)
	N0	4 (FN = 33.3%)	31 (TN = 88.6%)
		**Pathology CRM**	
		**Positive**	**Negative**
MRI CRM	Positive	1 (TP = 50.0%)	6 (FP = 13.3%)
	Negative	1 (FN = 50.0%)	39 (TN = 86.7%)

TP: True positive, TN: True negative, FN: False negative, FP: False positive.

## Data Availability

Data supporting the findings of this study are available from the corresponding author upon reasonable request.

## Abbreviations

The following abbreviations are used in this manuscript:

MRI	Magnetic resonance imaging
NAT	Neoadjuvant therapy
CRM	Circumferential resection margin
TME	Total mesorectal excision
EMVI	Extramural vascular invasion
